# Identification of a Novel Molecular Target for Alcohol Dependence

**DOI:** 10.1523/ENEURO.0255-18.2018

**Published:** 2018-07-09

**Authors:** Rosalind S.E. Carney

**Highlighted Research Paper:** Systemic and intra-habenular activation of the orphan G protein-coupled receptor GPR139 decreases compulsive-like alcohol drinking and hyperalgesia in alcohol-dependent rats, by, Jenni Kononoff, Marsida Kallupi, Adam Kimbrough, Dana Conlisk, Giordano de Guglielmo, and Olivier George

To treat the 15 million people in the US with alcohol use disorder (AUD), one may assume a good approach would be to identify the receptor associated with alcohol addiction and find a target compound that would block or activate the receptor. However, one needs to factor in the behavioral, emotional, and nociceptive changes associated with the rebound effects of alcohol withdrawal. People with AUD are aware that chronic, excessive alcohol intake has adverse consequences for their health, safety, and at times, the safety of others. But they can have a high compulsivity to drink alcohol, which can be overpowering, while suffering emotional dysregulation and a lowered pain threshold. Therefore, simultaneously mitigating several challenges associated with alcohol withdrawal could provide life-saving treatment for the ∼10% of people with AUD who seek treatment.

G protein–coupled receptors (GPCRs) are common targets for medical intervention. Those for which ligands have yet not been identified are called orphan GPCRs. In their eNeuro publication, Kononoff et al. (2018) examined if an orphan GPCR, GPR139, plays a role in alcohol dependence. They examined a part of the epithalamus, the habenula, that has high levels of GPR139 expression and is known to be involved in addiction and mood regulation (Matsuo et al., 2005).

Kononoff and colleagues used chronic intermittent exposure (CIE) to alcohol vapor combined with operant self-administration in rats. The CIE model is known to have predictive validity for AUD in humans (Macey et al., 1996; Heilig and Koob, 2007). A selective, brain-penetrant agonist, JNJ-63533054, was used to activate the GPR139 receptor.

**Figure 1.**
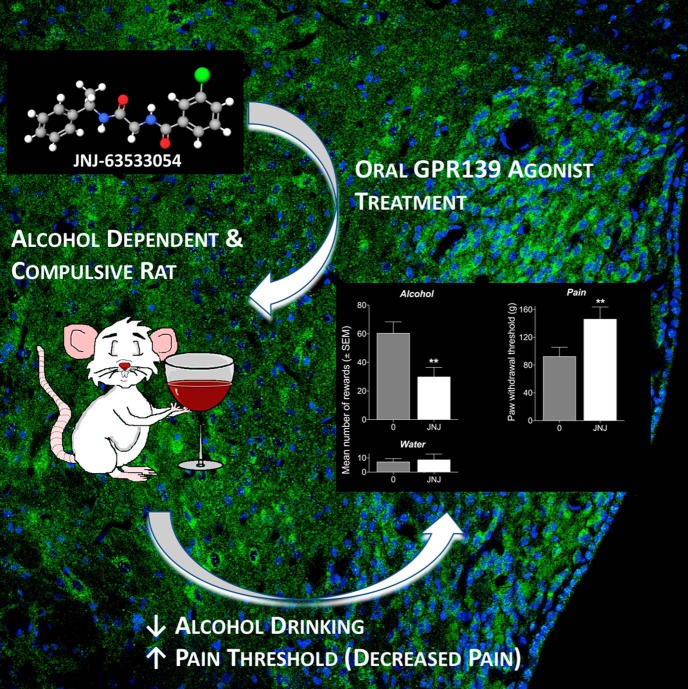
**GPR139 agonist JNJ-63533054 reduces alcohol dependence and increases the pain threshold.** Intra-habenular infusion of JNJ-63533054 decreased alcohol self-administration in alcohol-dependent rats compared to vehicle-treated rats. No effect on water intake was observed. During alcohol withdrawal, intra-habenular infusion of JNJ-63533054 increased paw withdrawal thresholds compared to vehicle-treated rats. (Figure courtesy of Jenni Kononoff.)

Systemic JNJ-63533054 administration significantly reduced alcohol self-administration in alcohol-dependent rats but did not have an effect in nondependent rats (Fig. 1). JNJ-63533054 did not affect saccharin or water administration in a separate cohort of rats that were made alcohol-dependent by CIE. These results indicate the specificity of JNJ-63533054 for reducing alcohol self-administration in alcohol-dependent rats.

To address the compulsive aspect of alcohol dependence, Kononoff and colleagues used the quinine adulteration test in two subgroups of alcohol-dependent rats: those with below- and above-median levels of alcohol intake. High-compulsive rats continue to self-administer alcohol adulterated with the adverse bitter taste of quinine, whereas low-compulsive rats decrease alcohol intake. When treated systemically with JNJ-63533054, high-compulsive rats significantly reduced their alcohol intake, whereas no effect was observed for low-compulsive rats. These findings again highlight the specificity of JNJ-63533054, and importantly, in the experimental group that needs an effect the most.

Alterations to pain threshold during alcohol withdrawal were tested using a paw withdrawal test. Systemic JNJ-63533054 treatment increased paw withdrawal thresholds compared to vehicle-treated alcohol-dependent rats (Fig. 1). This effect was even more significant for high-compulsive rats than low-compulsive rats. These data indicate that the GPR139 agonist could reverse the alteration in nociception (hyperalgesia) experienced during alcohol withdrawal.

To confirm the neuronal circuitry underlying the JNJ-63533054-mediated effect on hyperalgesia, Kononoff and colleagues microinjected JNJ-63533054 into either the habenula or interpeduncular nucleus (IPN) of alcohol-dependent rats. The IPN was selected as it is also involved in addiction, expresses GPR139, albeit at lower levels than the habenula, and has reciprocal connectivity with the habenula. These experiments confirmed that the habenula, and not the IPN, is the location where JNJ-63533054 exerts its effects on alcohol drinking and hyperalgesia.

Many researchers who uncover phenotypes of orphan GPCR function will next face the difficulties of identifying the endogenous ligand, but it is not particularly necessary in this case. Interestingly, several studies suggest that tryptophan, a precursor of serotonin, may be involved in GPR139-regulated alcohol dependence. Specifically, tryptophan depletion is associated with compulsive-like behavior in rats (Merchan et al., 2017), alcohol abuse correlates with low plasma concentrations of tryptophan in humans (Virkkunen and Linnoila, 1990), and brain tryptophan levels are reduced during alcohol withdrawal (Bano et al., 1996). These observations lend support to the hypothesis that activating GPR139 may help counter some of the adverse aspects of alcohol dependence.

Currently, Professor Olivier George and his lab (The Scripps Research Institute, La Jolla, CA) are focused on looking for other agonists and antagonists for GPR139 that may have even better potency and efficacy on pain, alcohol, and other drug addictions. Recent rodent and human transcriptome data found a cluster of highly expressed habenular genes that include the µ opioid receptor (Boulos et al. 2017). The goal of the George lab is to find a combination of compounds that will alleviate alcohol withdrawal systems as much as possible and prevent excessive drinking to give someone with AUD the best chance to live an alcohol-free life or at least reduce harmful, compulsive drinking.

A potential for GPR139 to be part of AUD treatment seems optimistic because of certain features of both the receptor and the agonist. GPR139 is highly expressed in very specific regions of the central nervous system that are involved in addiction and has low levels of expression in the rest of the body, so adverse effects during treatment are not expected. JNJ-6353305A, though given orally, survives passage through the gut, crosses the blood–brain barrier, and has sufficient bioavailability to be effective in the brain, providing strong evidence that GPR139 is a good druggable target.

Overall, the identification of this novel molecular target associated with alcohol addiction is an important step in the establishment of a successful treatment regimen for people with AUD. And fortunately, the next steps of this study are technically feasible and could reveal useful information about other areas of addiction.
